# Supplementary material for: Global hypo-methylation in a proportion of glioblastoma enriched for an astrocytic signature is associated with increased invasion and altered immune landscape
Source: eLife. 2022 Nov 22;11:e77335. doi: 10.7554/eLife.77335 (PMC9681209; doi:10.7554/eLife.77335)
Supplement: Figure 2—source data 1. [file elife-77335-fig2-data1.zip › Figure_2_source_data_1/Figure_2I_J/homerResults/motif21.similar.html]

motif21

## Information for motif21

C
G
T
A
A
C
T
G
C
G
A
T
C
A
G
T
A
T
C
G
T
C
G
A
A
G
T
C
G
A
T
C
  
Reverse Opposite:  

C
T
A
G
A
C
T
G
A
C
G
T
A
T
G
C
G
C
T
A
C
G
T
A
A
G
T
C
A
C
G
T
  

|  |  |
| --- | --- |
| p-value: | 1e-33 |
| log p-value: | -7.772e+01 |
| Information Content per bp: | 1.790 |
| Number of Target Sequences with motif | 52.0 |
| Percentage of Target Sequences with motif | 7.16% |
| Number of Background Sequences with motif | 2.2 |
| Percentage of Background Sequences with motif | 0.77% |
| Average Position of motif in Targets | 99.4 +/- 53.2bp |
| Average Position of motif in Background | 118.7 +/- 39.2bp |
| Strand Bias (log2 ratio + to - strand density) | -0.6 |
| Multiplicity (# of sites on avg that occur together) | 1.04 |
| Motif File: | file (matrix) reverse opposite |

### Similar de novo motifs found

|  |  |  |  |  |  |  |  |
| --- | --- | --- | --- | --- | --- | --- | --- |
| Rank | Match Score | Redundant Motif | P-value | log P-value | % of Targets | % of Background | Motif file |
| 1 | 0.657 | C A T G C A G T A G C T T G C A G C T A G A T C G C T A A T C G | 1e-16 | -38.102990 | 9.37% | 2.87% | motif file (matrix) |
| 2 | 0.783 | A C T G A C G T A T G C C G T A C G T A A G T C | 1e-11 | -25.555717 | 16.80% | 9.19% | motif file (matrix) |
